# Supplementary material for: HMGB1/NF-κB Axis, IL-8, and Cuproptosis Contribute to Cisplatin-Induced Testicular Injury: Protective Potential Effect of Thymol
Source: Biomolecules. 2025 Nov 14;15(11):1595. doi: 10.3390/biom15111595 (PMC12650315; doi:10.3390/biom15111595)
Supplement: Supplementary file 1 [file biomolecules-15-01595-s001.zip › biomolecules-3962200 Figure S1.pdf]

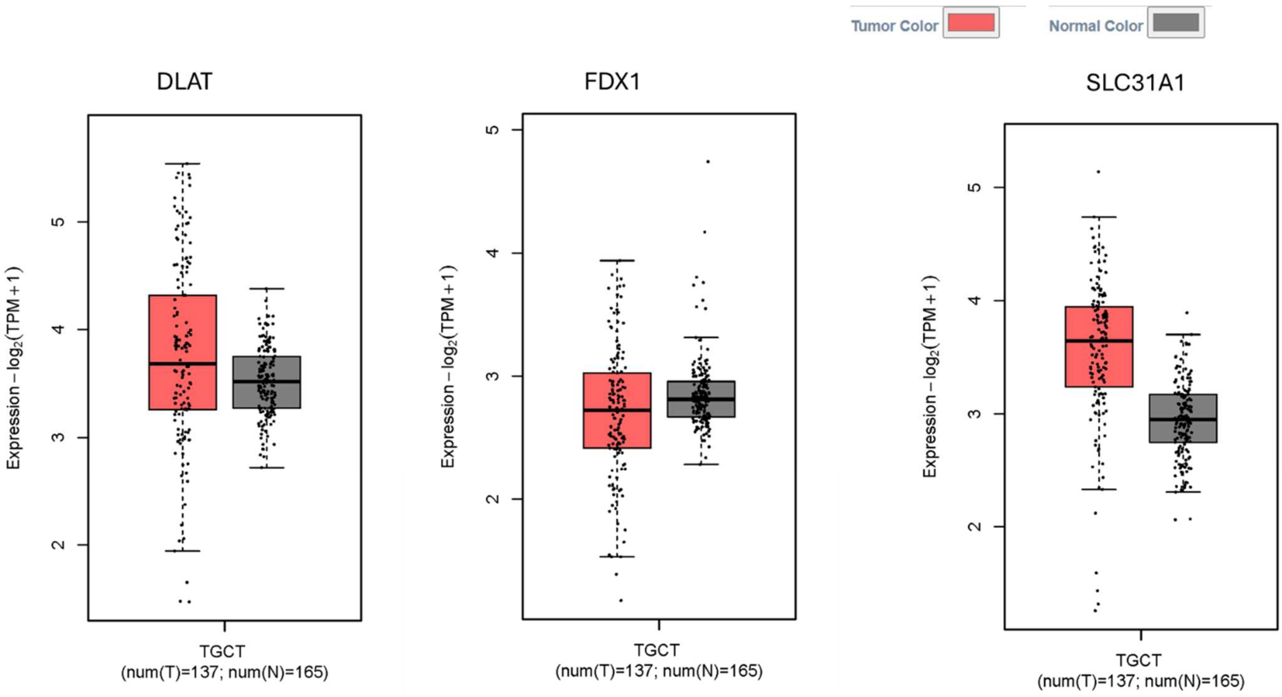

Figure S1. Expression Profile of cuproptosis-related genes in TGCT and Normal tissues. Box Plot representing the expression of FDX1(A), DLAT (B), and LIAS (C) in Testicular Tumor (T, n=137) and normal samples (N, n=165). *P*-value cutoff was set to 0.01, log2FC (fold change) cutoff =1. Data are expressed as log_2(TPM(Transcripts Per Million)+1) and presented as Median ±interquartile range.
