# Supplementary figures and images for: HMGB1/NF-κB Axis, IL-8, and Cuproptosis Contribute to Cisplatin-Induced Testicular Injury: Protective Potential Effect of Thymol
Source: Biomolecules. 2025 Nov 14;15(11):1595. doi: 10.3390/biom15111595 (PMC12650315; doi:10.3390/biom15111595)

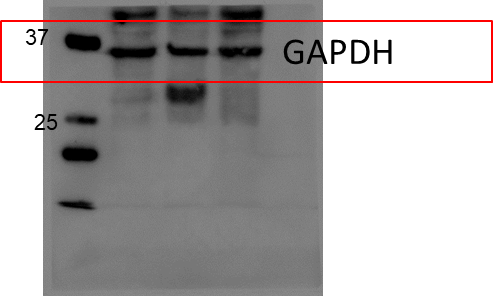

Supplement: Supplementary file 1 [file biomolecules-15-01595-s001.zip › GAPDH Original .tif]

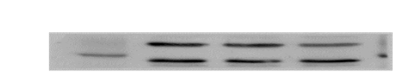

Supplement: Supplementary file 1 [file biomolecules-15-01595-s001.zip › GAPDH.tif]

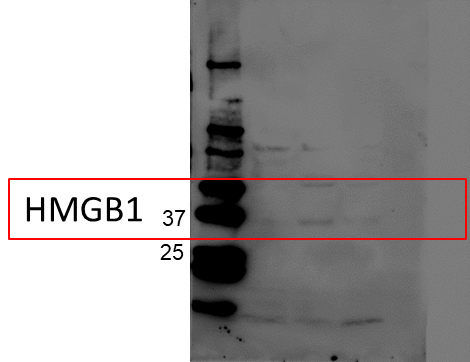

Supplement: Supplementary file 1 [file biomolecules-15-01595-s001.zip › Western Original Blot for figure 5 (a) .tif]

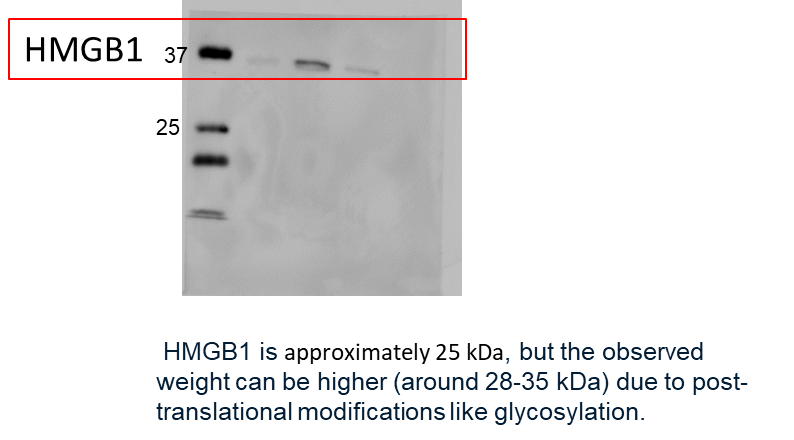

Supplement: Supplementary file 1 [file biomolecules-15-01595-s001.zip › Western Original Blot for figure 5 (b) .tif]
